# Use of Computer Digital Techniques and Modern Materials in Dental Technology in Restoration: A Caries-Damaged Smile in a Teenage Patient

**DOI:** 10.3390/jcm13185353

**Published:** 2024-09-10

**Authors:** Katarzyna Zaborowicz, Marcel Firlej, Ewa Firlej, Maciej Zaborowicz, Kamil Bystrzycki, Barbara Biedziak

**Affiliations:** 1 Department of Orthodontics and Facial Malformations, Poznan University of Medical Sciences, Bukowska 70, 60-812 Poznań, Poland; marcel-firlej@wp.pl (M.F.); efirlej@ump.edu.pl (E.F.); biedziak@ump.edu.pl (B.B.); 2 Department of Biosystems Engineering, Poznan University of Life Sciences, Wojska Polskiego 50, 60-637 Poznań, Poland; 3 Poznan University of Medical Sciences, Collegium Maius ul. Fredry 10, 61-701 Poznań, Poland; kamil.bystrzycki@wp.pl

**Keywords:** interdisciplinary treatment, pediatric dentistry, prosthetic restorations, 3D-printed composite crowns

## Abstract

Prosthodontic treatment of developmental age patients presents a significant challenge to the dentist. The growth and development of the stomatognathic system must be considered in treatment planning. Temporary prosthetic restorations must be regularly inspected and recemented, and final prosthetic restoration should not be delivered until the growth of the body is complete. In addition, due to the complex nature of morphological and functional disorders during the developmental period, simultaneous prosthetic and orthodontic treatment may be required. The case presented in this article is a 16-year-old boy with severe tooth destruction caused by untreated caries disease and poor oral hygiene. The patient required conservative, endodontic, and surgical treatment to restore the occlusion and aesthetics to allow the proper development of the masticatory organ. This article also presents the treatment case of a young patient with damaged crowns in the upper arch, which were restored with standard root–crown posts and cores and temporary 3D-printed composite crowns.

## 1. Introduction

The prosthetic treatment of children and adolescents is always problematic. The growth of the body and changing dimensions of the craniofacial region make most permanent prosthetic restorations contraindicated. Also, any removable prosthetic restorations must be frequently replaced without destroying tooth structure in order not to inhibit the correct growth of the alveolar process of the maxilla and the alveolar region of the mandible. The most important rule in the prosthetic treatment of young patients is to choose such structures that will not interfere with the development of the stomatognathic system. It should be remembered that the loss of teeth in the developmental age leads to such disorders as masticatory function disorder, swallowing disorder, pronunciation disorder, the growth inhibition of the facial part of the skull, lowering of occlusion height, mandibular displacement, and changes in facial features. As a consequence, self-esteem may also be significantly lowered. Prosthetic treatment for children and adolescents is aimed to at preventing disorders caused by missing teeth, the improvement of appearance, well-being, and improvement of chewing function [1].

The decision of prosthetic restoration depends on the age of the patient, the cause of tooth loss, and the presence of orthodontic and developmental defects. The main indications for prosthetic treatment are congenital deficiencies in dentition, developmental defects, and post-traumatic or carious damage to teeth.

Some genetic syndromes are associated with missing tooth buds, the presence of persistent deciduous teeth and retained teeth, and abnormal dental hard tissue structure. Patients born with such syndromes require prosthetic treatment from an early age [2].

The main challenge for dentists is to plan a treatment that will not disturb the development of the stomatognathic system while improving the patient’s chewing function, pronunciation, and appearance. In patients with ectodermal dysplasia or facial clefts, in addition to missing tooth buds, reduced bone tissue in the alveolar process of the maxilla or the alveolar region of the mandible is problematic. As a result, the shortening and reshaping of the dental arches also occurs, and in patients with oligodontia and anodontia, there is a shortening of the lower facial height [3,4,5].

Prosthetic treatment of patients with ectodermal dysplasia depends on the severity of stomatognathic changes. In addition to prosthetic treatment, they require interdisciplinary treatment and mainly orthodontic treatment to improve intermaxillary interrelations [6,7,8].

Patients with a cleft palate also require prosthodontic/orthodontic therapy. As a result of missing or abnormal tooth buds and reduced bone in the alveolar process, the underdevelopment of the jaw occurs. They may experience micrognathia, anterior overbite, open bite, and deep bite. An additional challenge is the presence of a cleft fissure penetrating into the nasal cavity [9,10].

At developmental age, due to the continuous growth and development of the alveolar process of the maxilla and the alveolar part of the mandible, removable prostheses are the most commonly used prosthetic restorations. It is recommended to start prosthetic treatment in early school-aged children in order to improve the patient’s psychosocial development. Dentures should be regularly inspected and replaced. It is recommended to replace the denture every 8–10 months in children under 11 years of age, every 18 months in children between 11 and 15 years of age, and every 24 months in children aged 15–18 years [11,12].

In recent years, digitally designed restorative methods have become increasingly popular. This allows the dentist to create a precise plan for the rehabilitation of the smile. In addition, in a patient of developmental age, it allows prosthetic restorations to be designed in a way that will not inhibit the development of the stomatognathic system. Many areas of digital dentistry are available, such as computer-aided design/computer-aided manufacturing (CAD/CAM) and digital smile design. Temporary restorations are common solutions in dentistry, and materials used for long-term restoration should have high durability [13]. In recent years, 3D printing and the use of additive technology have gained popularity for the fabrication of temporary crowns. Digital light processing technology (DLP) provides good quality and reproducibility with high accuracy [14]. Indeed, 3D-printed temporary crowns are commonly used in prosthetics as a low-cost alternative to conventionally cured provisional dental materials [15]. Stable 3D-printed models of patient’s teeth may also be required for the final processing of restoration. Materials should be resistant to wear and also have high indentation hardness [16]. Resins used for long-term temporary restorations should also be resistant to bending and have good shape stability [17].

In exceptional cases, such as oligodontics or anodontics, treatment with intraosseous implants as a retention element for removable dentures is allowed. The implants should be implanted in the space between the foramen ovale. The performance of a lower denture based on two implants provides good retention [18,19].

The aim of this case report is to present a case of the treatment of an adolescent patient with extensive destruction of hard tissues in the teeth due to caries. The patient’s diet comprised mainly sugary snacks and sweetened, fizzy drinks. They caused teeth damage and caries development on weakened dental hard tissue [20].

## 2. Case Report

**Figure 1:**
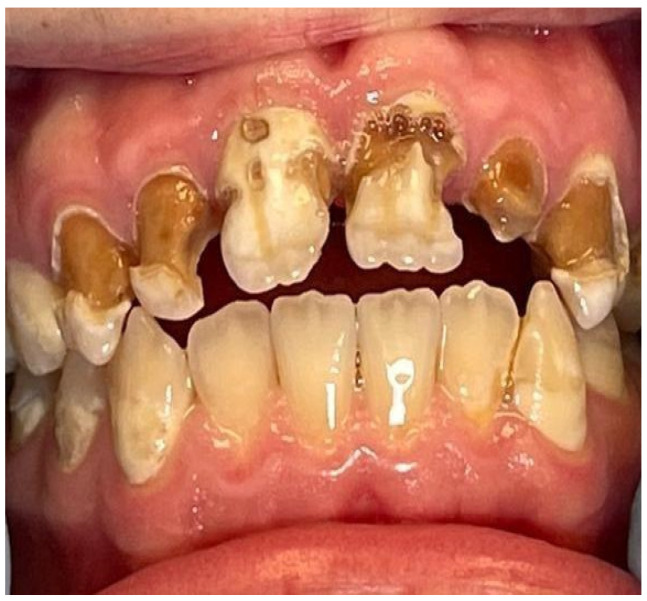
Patient’s dentition before treatment with extensive visible caries in the anterior segment—view of the smile.

**Figure 2:**
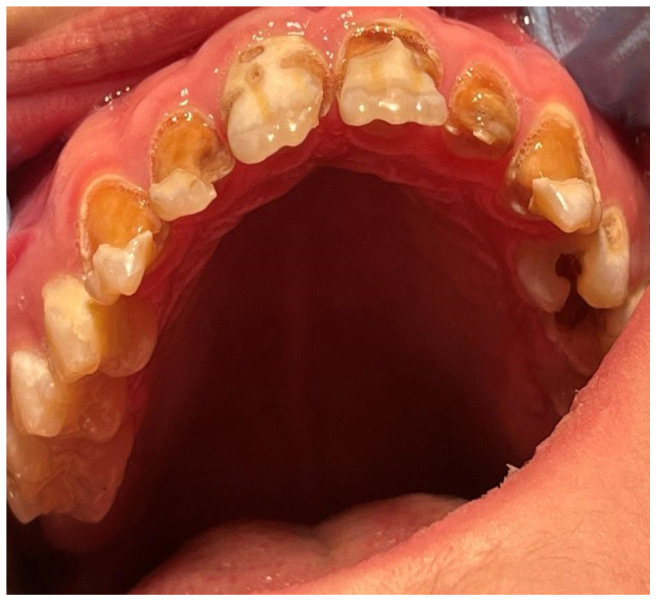
Patient’s dentition before treatment—view of occlusal surface of teeth in the maxilla.

**Figure 3:**
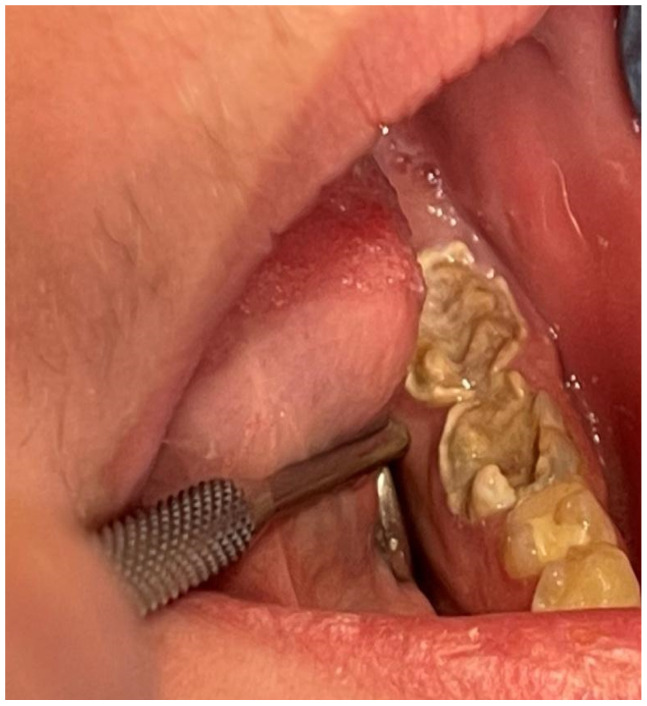
Patient’s dentition before treatment with visible destruction of permanent molar crowns—view of lateral teeth in the mandible.

**Figure 4:**
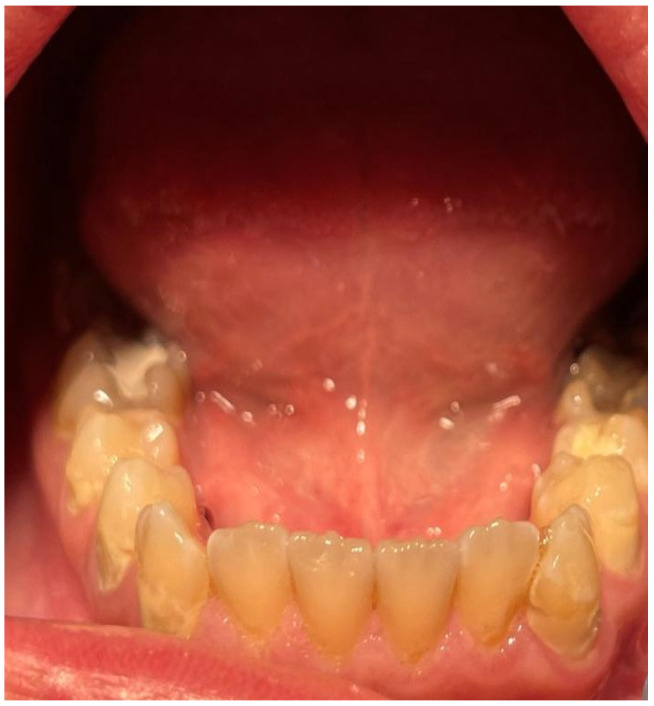
Patient’s dentition before treatment—view of teeth in the mandible.

**Figure 5:**
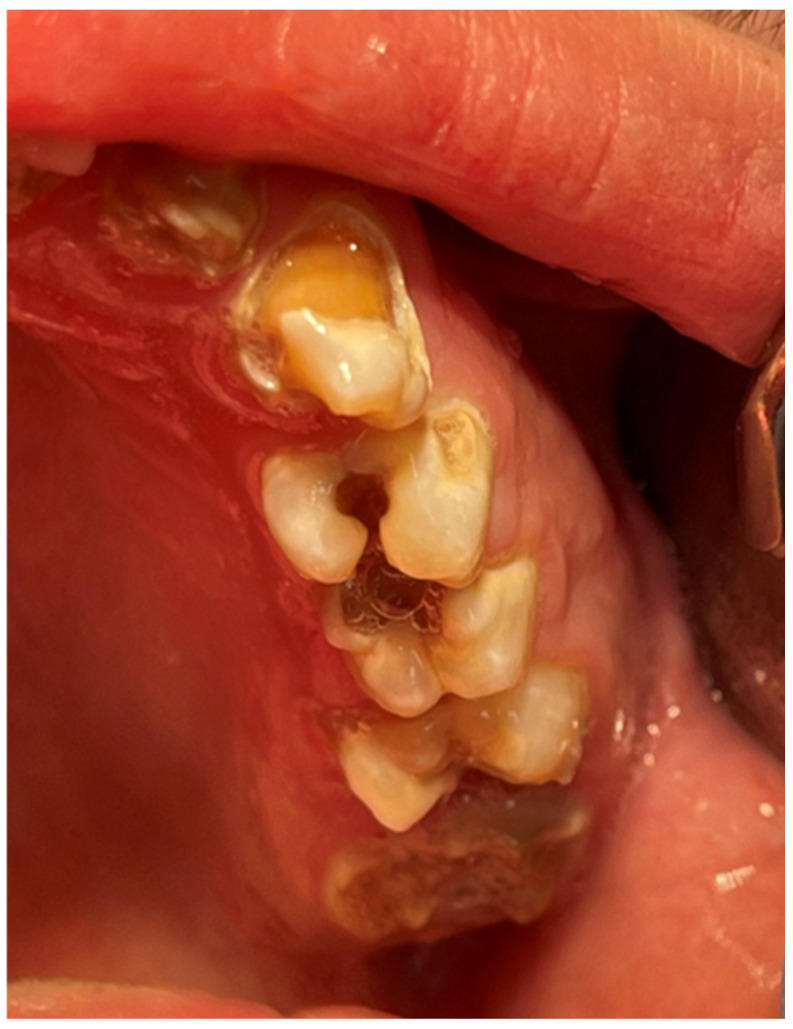
Patient’s dentition before treatment with visible destruction of tooth crowns in the lateral segment—view of lateral teeth in the maxilla.

A 16-year-old patient came to the Pediatric and Adolescent Dentistry Clinic of the University Center for Dentistry and Specialized Medicine in Poznań for oral examination and treatment. According to the interview, the patient did not suffer from any systemic diseases and did not take any permanent medication. The last dental treatment was performed many years ago. On the day of the first appointment, he complained about occasional toothaches during cold drinks consumption. The patient was also unsatisfied with the appearance of his anterior teeth (Figure 1, Figure 2, Figure 3, Figure 4 and Figure 5).

**Figure 6:**
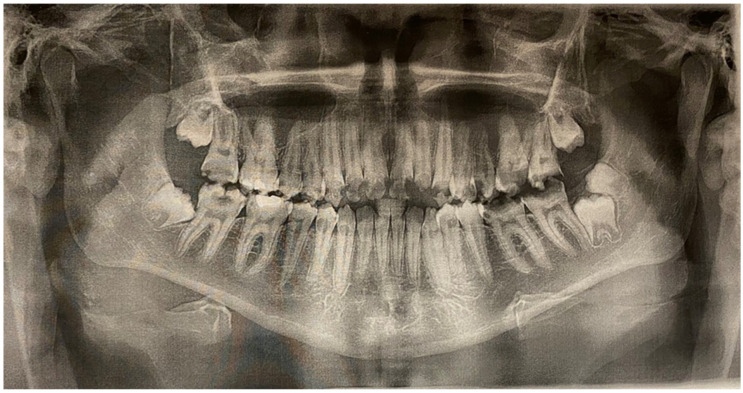
Pantomographic picture of the patient.

An oral examination with hygiene instruction and dietary recommendations was performed. The patient brushed his teeth only once a day, and his diet was dominated by sugary snacks and sweetened soft drinks. The patient was referred for a pantomographic radiograph (Figure 6).

The oral examination qualified teeth 17, 26, 27, 36, 37, and 47, and an additional tooth behind the wisdom tooth on the left side in the mandible for extraction. The following teeth were qualified for conservative treatment: 14, 15, 16, 33, 34, 35, 43, 44, 45, and 46. In teeth 13, 12, 11, 21, 22, 23, 24, and 25, due to very large carious destruction of hard tissues reaching the pulp chamber, root canal treatment and prosthetic restoration were planned.

An orthodontic and prosthodontic consultation was provided. The orthodontic examination demonstrated that the patient had an open bite with an elongated lower facial segment. Due to the very severe destruction of the crowns of the molars, the biting of food was mainly performed on the premolars.

The young age of the patient and the ongoing growth of his body limited the possibilities of providing prosthetic work to the maxilla. Additionally, the poor economic situation of the family did not allow for expensive treatment. Two options for prosthetic treatment were proposed. The first was to construct a removable partial denture placed on endodontically treated tooth roots in the maxilla. The second proposal was a prosthetic restoration using standard root–crown posts and cores and temporary composite crowns designed using 3D software. This technique allows the dentist to make easy changes in the 3D software and inexpensive and fast production. It also eliminates the need for inaccurate free-hand composite bonding [21] and eliminates the need for plaster model production and mounting in standard articulators, which is also time-consuming and may be inaccurate [22].

The patient and his parents decided on a treatment option with posts, cores, and composite crowns. First, teeth 14, 15, 16, 33, 34, 35, 43, 44, 45, and 46 were restored. Endodontic and initial restorative treatment was carried out by K.Z., and final conservative treatment and prosthodontics with CAD/CAM production was conducted by M.F.

In the next step, teeth 13, 12, 11, 21, 22, 23, 24, and 25 were treated with root canal therapy. The canals were prepared by machine using a Reciproc device (VDW), with the rinsing of the canals with NaOCl 5.25% solution, H_2_O_2_ and NaCl. The length of the canals was measured using an endometer (VDW), and a radiograph with a gutta-percha stud was taken to verify the measurement. The canals were filled using a single Reciproc gutta-percha stud and AHplus sealer (Dentsply Sirona Dentsply DeTrey GmbH De-Trey-Str. 1 78467, Konstanz, Germany). The seal of the canal filling was checked on dental radiographs. The very severe carious destruction of the crowns of teeth 13, 12, 21, 22, 23, and 24 meant that there was very little original crown on the surface, providing a very small ferrule effect for a future crown.

**Figure 7:**
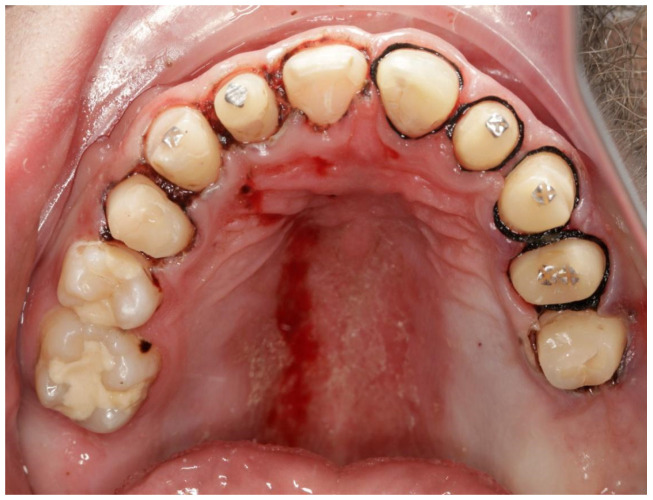
Patient’s dentition after endodontic treatment, with standard crown–root posts prepared for crowns.

**Figure 8:**
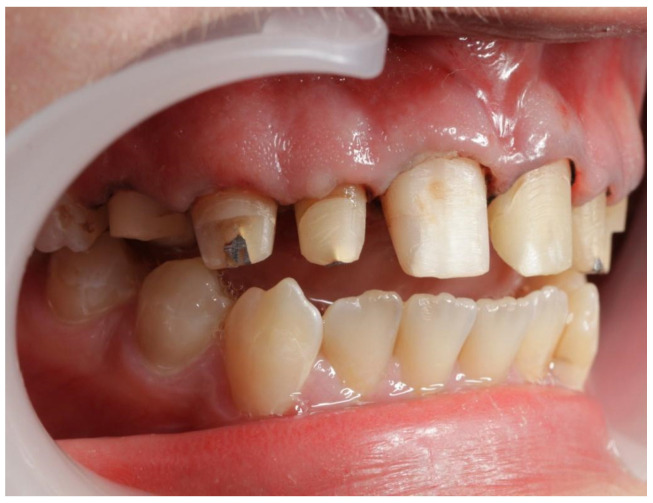
Patient’s teeth prepared for crowns—view of lateral teeth.

**Figure 9:**
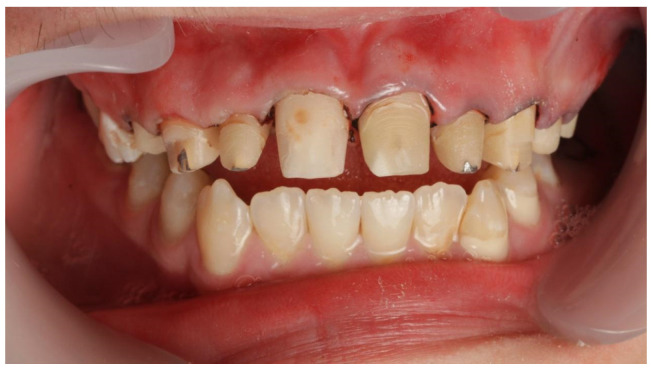
Patient’s teeth prepared for crowns—view of anterior teeth.

The treated roots were then supplied with standard crown–root posts (Nordin). The posts were cemented in place using dual cure Activa BioACTIVE-Restorative (PULPDENT), which was also the core material. The rest of the teeth, which had previously undergone root canal treatment, were able to be restored with composite material and prepared for the composite crowns. Due to the young age of the patient and severe destruction of original teeth tissue, the teeth were prepared vertically using the shoulderless and edgeless technique with a preparation limit of less than 0.5–1 mm subgingival (Figure 7, Figure 8 and Figure 9) [23]. The prosthetic field prepared in this way was scanned with a Trios 3shape intraoral scanner. Until the next visit, the teeth were protected with acrylic temporary crowns.

**Figure 10:**
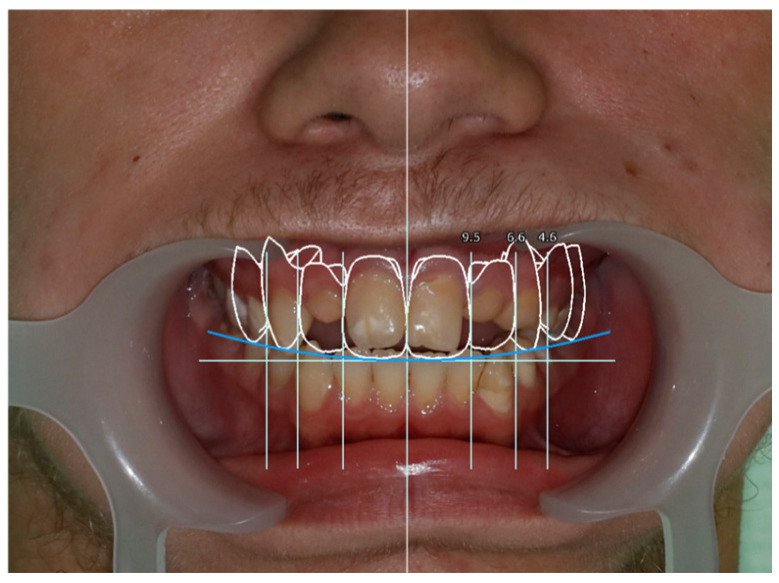
Digital smile design.

**Figure 11:**
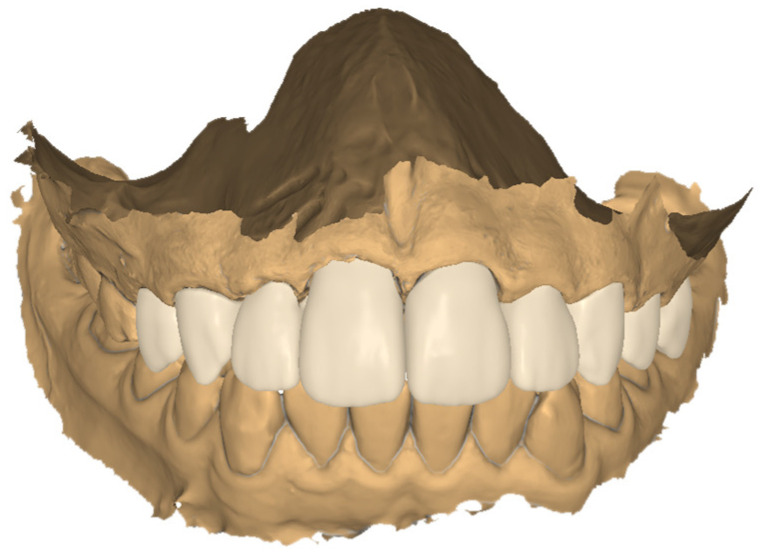
Design of the crown superimposed on the tooth scan.

**Figure 12:**
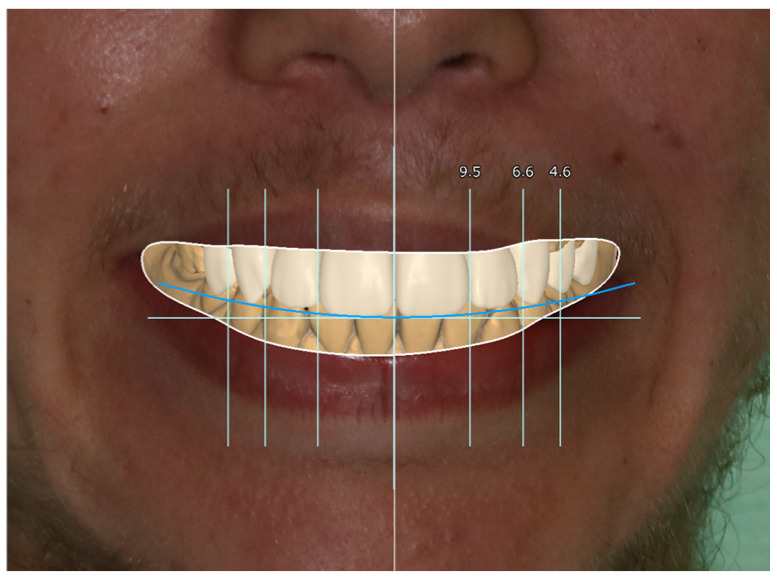
Design of the crown superimposed on patient’s photo.

**Figure 13:**
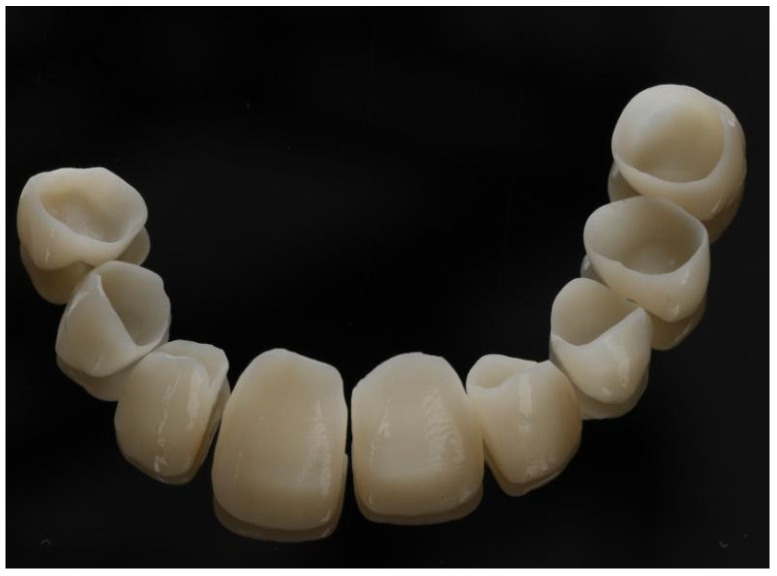
Prepared crowns prior to seating on the teeth.

**Figure 14:**
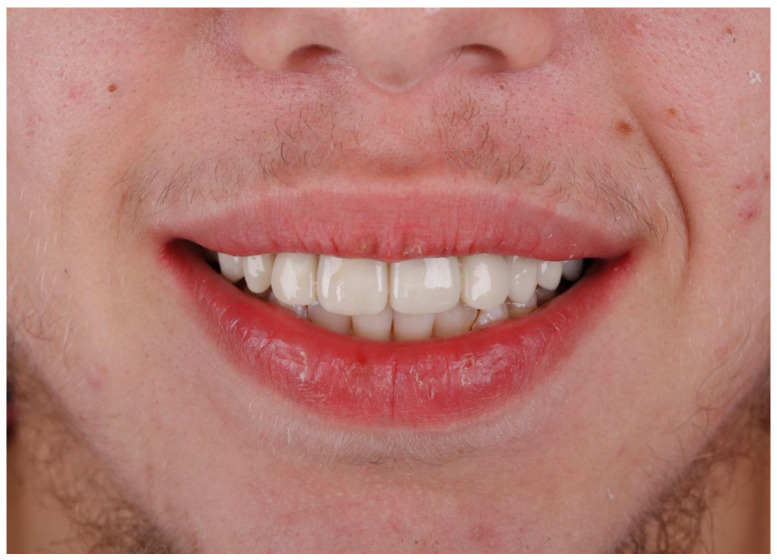
Patient’s long-term temporary dentition after treatment—anterior view of the smile.

**Figure 15:**
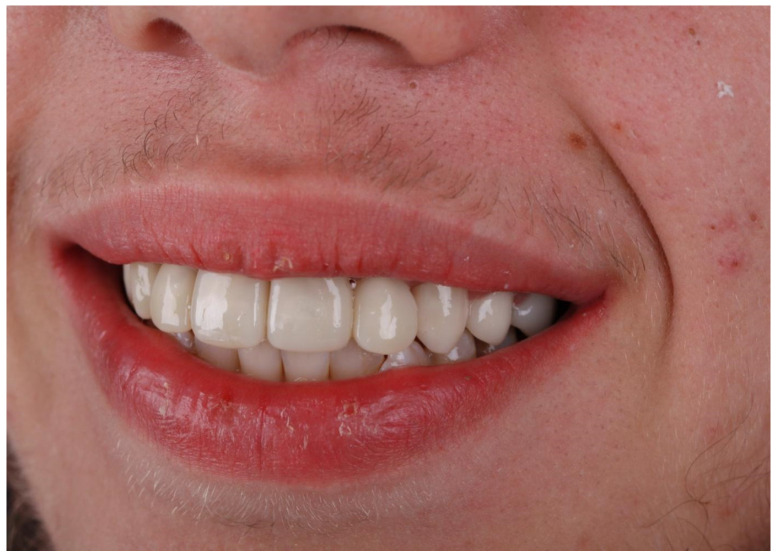
Patient’s long-term temporary dentition after treatment—lateral view of the smile.

In the next step, jaw models were printed in the Phrozen MINI4k 3D printer (Phrozen 3D Printers Europe, Wasaweg 3, Groningen, The Netherlands). Digital smile design was performed using patients’ photographs for correct teeth sizes and best placement esthetic lines according to the patient’s face (the width of marked sections in millimeters) (Figure 10, Figure 11 and Figure 12). The correct anatomy of the last tooth in the arch (in this case, a premolar) was restored, which was adequate for the patient’s young age, as well as the premolar on the other side of the arch. This height determined the maximum height of the remaining teeth to be occluded, where the restoration of contacts was possible. Exocad was used for CAD (computer-aided design) and crown shape design, and a Phrozen 3D printer was used for CAM (computer-aided manufacturing) crown printing. Nextdent MFH C&B (Nextdent) was used for crown printing due to its good stability in saliva [17,24]. After printing, remaining resin excesses were removed in the Anycubic Wash and Cure Machine (Anycubic) and alcohol IPA 99% solution [16]. After drying, crowns were finally cured in the same device. Then, 3D printing supports were cut off, and for the final shining and sealing effect, a transparent glaze Optiglaze (GC) was used (Figure 13, Figure 14 and Figure 15). During the next appointment, crowns were cemented, and the patient was instructed about the diet, oral hygiene, and control visits that were scheduled.

The final stage of the complex treatment was the extraction of teeth 17, 26, 27, 36, 37, 39, and 47. Because of the elongated lower facial segment, the young age of the patient, and the fact that the patient had been biting his food with the premolars for many years, no prosthetic reconstruction of the molars was planned yet. Orthodontic treatment for the lower arch was recommended when oral hygiene was improved.

A control visit 6 months later showed that the crowns were stable despite the patient’s bad hygiene, and the patient was satisfied with the treatment.

## 3. Discussion

Prosthetic treatment in young patients requires a case-by-case approach and multiple follow-up visits. Prosthetic restorations placed at a young age are temporary solutions and need to be replaced with definitive restorations after growth has ended. Nevertheless, in many cases, starting treatment at a young age is essential. Missing teeth can cause aesthetic, speech, and eating disorders. Patients who have missing teeth due to genetic defects or the loss of hard tooth tissue due to caries disease require prosthetic restoration to improve their quality of life. The psychological aspect is very important in young people [25,26].

It is very common for dental disorders to be accompanied by malocclusion. In such cases, interdisciplinary treatment is necessary, and prosthetic restorations often also serve as orthodontic appliances. Prosthetic rehabilitation also helps to improve tongue position and eliminate persistent infantile types of swallowing [6]. The accuracy of digital technology allows the fabrication of the proper individual anatomy of patients teeth in CAM software (DentalCAD 3.1) [27]. These restorations can be inexpensively replaced or repaired in the case of material wear or fracture.

Patients of developmental age who may require precise prosthodontic treatment are those who have lost hard tooth tissue or entire teeth due to trauma or by carious disease. Prosthetic restorations in the lateral segment are provided to maintain occlusal height, retain space for developing tooth buds, and improve chewing function. Anterior restorations should also improve aesthetics [28,29].

The other group of patients who require oral prosthetic work are patients with developmental defects. This group includes mainly those with a cleft lip and palate and ectodermal dysplasia. Patients with these disorders are a major challenge for the clinician. When planning prosthetic treatment in such patients, it is important to remember that the alveolar processes of the maxilla and the alveolar part of the mandible are malformed, and the teeth in the oral cavity have abnormal structure and dimensions [30,31].

The use of digital technology supports dentists’ efforts to ensure that the patient receives the best possible treatment in the most comfortable environment. Digital radiographs, CAD/CAM technology, digital smile design, digital shade matching, as well as digital facial arches, and virtual articulators are examples of technologies used in dental practice to design and create restorations for young patients. These technologies are particularly valuable because they allow prosthetic restorations to be made without interfering with the patient’s growth [32].

Temporary restorations in the oral environment are exposed to different aging factors. They are exposed to the temperature of the oral cavity and also to the saliva for a period of the pre-treatment time. Some authors report that the polymer matrix structure after water acts leadsto relaxation and a reduction in material stiffness [33]. This mechanism has not been well investigated, but it is suggested that the polymer structure with ester, amide, and urea groups might increase material degradation [34]. This intensity of the material degradation is also dependent on the surface condition and the proportion of additional substances, such as filler particles with different sizes [35]. Long-term storage in artificial saliva shows changes on the indentation hardness and the abrasive wear of different composite materials used for temporary CAD/CAM restorations [24]. Most properties of resins used for 3D-printed temporary restorations have lower stiffness, elasticity, degradation rate, and higher wear after aging for 6 months in artificial saliva. However, some materials like the one used for this case presentation (Nextdent Crown & Bridge MFH) showed a remarkable increase in hardness after aging, which was accompanied by a significant decrease in the modulus of elasticity. The increase in coefficient H2/E of the Nextdent MFH C&B material after aging, caused mainly by a significant increase in indentation hardness, may be related to its structure. It was the material with the most complex composition among the tested materials, classified as a micro-hybrid material with microfille [36].

In the case described, the decision was made to restore the teeth with posts and cores and temporary 3D-printed composite crowns, which were designed in Exocad and printed in the Phrozen 3D printer. This procedure was due to the fact that the patient’s dental hard tissue destruction was so severe that many teeth required endodontic treatment, and the doctors wanted to strengthen the teeth with posts and cores.

In this case, other therapeutic options were also possible and were presented to the patient at the first visit. One possibility was to create a removable partial denture placed on endodontically treated tooth roots in the maxilla. Another possibility was also the removal of teeth from which hard tissue destruction caused by caries reached the pulp and the creation of a partial denture. Teeth could also be restored with composite material using fiberglass inlays. However, in this case, the clinicians were keen to make a restoration that preserved as much of the tooth’s hard tissue as possible and to strengthen endodontically treated teeth. The digital smile design allowed crowns to be designed very precisely and in a way that is aesthetically pleasing. Given the patient’s previous lack of oral hygiene and a diet consisting mainly of sugary snacks and sweetened, fizzy drinks, the creation of composite crowns increases the chance of long-term retention of the restoration. Furthermore, the patient had an open bite with an elongated lower facial segment. The digital crown design improved the appearance of the smile by reducing the underbite gap between the front teeth. Temporary prosthetic rehabilitation in adolescent growing patients require frequent control visits. Oral hygiene education is crucial to maintain healthy soft tissue around the teeth and hard tooth tissue supporting prosthetic crowns. The patient was educated and informed about the importance of diet and oral hygiene during the temporary crown rehabilitation process. The limitation of this kind of temporary treatment is the patients’ and parents’ responsibility. Although 3D printing technology for temporary crown fabrication is a promising, fast, and inexpensive technology, it still requires further research and longer follow-ups.

## 4. Conclusions

In the presented case, a 16-year-old patient had prosthetic treatment due to the severe destruction of teeth by carious disease and the overconsumption of sugary snacks and sweetened, fizzy drinks, resulting in the weakness of the dental hard tissue.

The treatment of an adolescent patient with massive dental hard tissue destruction requires a multidisciplinary approach so that it does not interfere with the development of the stomatognathic system. The restoration must be permanent enough to last until the patient reaches an age at which definitive restorations can be carried out [36,37]. The mechanical properties of 3D-printed temporary crowns show that they can be an alternative to other types of restoration in growing patients; however, due to their mechanical properties, they often require control visits, especially for the patients with bad oral hygiene. The technological development of 3D-printed materials in this field may replace traditional methods in the future for other types of temporary restorations.

## Data Availability

This research did not require any additional data.
